# Comprehensive Insights into Chondroblastoma Metastasis: Metastatic Patterns and Therapeutic Approaches

**DOI:** 10.3390/cancers16122283

**Published:** 2024-06-20

**Authors:** Ramy Samargandi, Abrar Bafail, Louis-Romée Le Nail, Julien Berhouet

**Affiliations:** 1 Service de Chirurgie Orthopédique et Traumatologique, Centre Hospitalier Régional Universitaire (CHRU) de Tours, 1C Avenue de la République, 37170 Chambray-les-Tours, France; lr.lenail@chu-tours.fr (L.-R.L.N.); julien.berhouet@gmail.com (J.B.); 2 Department of Orthopedic Surgery, Faculty of Medicine, University of Jeddah, Jeddah 23218, Saudi Arabia; 3 Service de Médecine Nucléaire, Centre Hospitalier Régional Universitaire (CHRU) de Tours, 1C Avenue de la République, 37170 Chambray-les-Tours, France; abrar.a.b@hotmail.com

**Keywords:** chondroblastoma, denosumab, metastasis, bone tumors, musculoskeletal oncology

## Abstract

**Simple Summary:**

Chondroblastoma is a rare benign aggressive primary bone tumor, with susceptibility to local recurrence and metastasis, mainly to the lungs. However, various metastatic locations have been described, such as bone, soft tissue, and liver. Metastatic chondroblastoma treatment involves various approaches including surgical resection, radiotherapy, chemotherapy, and palliative treatment. The expression of RANKL in chondroblastoma cells has opened new avenues for treatment with denosumab, which shows effectiveness in controlling both local and metastatic tumors. Hence, there is a greater need to enhance our understanding of this rare tumor’s biology and refine therapeutic approaches to improve patient outcomes, reduce recurrence rates, and better manage metastatic cases.

**Abstract:**

Chondroblastoma metastasis, though rare, represents a clinically significant and notably important aspect of bone tumors. Understanding its epidemiological characteristics, pathological features, and treatment modalities, despite its infrequency, is imperative for comprehensive patient management. This review aims to elucidate the epidemiology, molecular mechanisms, diagnostic challenges, and therapeutic strategies associated with chondroblastoma metastasis. The patterns, prognostic factors, and treatment outcomes were explored through an analysis of case studies and clinical reports. Notably, we highlighted emerging therapeutic perspectives aimed at improving patient outcomes. To the best of our knowledge, there has been no previous review addressing these matters cumulatively, highlighting a significant gap in the existing scholarly literature. By shedding light on the nuances of chondroblastoma metastasis, this review contributes to the advancement of knowledge in this field and informs clinical decision-making for improved patient care.

## 1. Introduction

Chondroblastoma is a rare primary bone tumor that primarily affects the epiphyseal regions of long bones [1,2]. Histologically, chondroblastomas exhibit a characteristic appearance, with clusters of polygonal or rounded chondroblast-like cells embedded within a hyalinized or myxoid matrix [3,4]. While generally considered benign, chondroblastomas have the potential for local recurrence and can, infrequently, metastasize [5]. The World Health Organization (WHO) classifies chondroblastoma as an intermediate (rarely metastasizing) tumor [6]. Grading by the WHO primarily considers the histological features of the tumor, including cellularity, nuclear pleomorphism, mitotic activity, and necrosis. Chondroblastoma can manifest across all age groups, although it predominantly emerges during adolescence and early adulthood. Additionally, males are more susceptible than females, with a male-to-female ratio of 2:1 [3,7,8]. The incidence rate of chondroblastoma fluctuates across diverse populations and geographical areas. Although this tumor can manifest in any bone, it predominantly emerges in the epiphyseal regions of long bones, notably in the proximal humerus, proximal femur apophysis, and proximal tibia [9,10,11]. Other frequent locations include the bones of the hands or feet, such as the talus, phalanges, and metatarsal bones. Additionally, flat bones are also susceptible to this tumor, with documented cases in the pelvis, ribs, patella, sternum, clavicle, and vertebrae [12,13,14,15]. Although less commonly observed, tumors can also originate in the skull and facial bones, including the temporal bone and mandible [16]. The primary symptom of chondroblastoma at initial presentation is often pain, which is usually mild and worsens gradually. Initially, patients may dismiss this pain as resulting from a minor injury. If the lesion is located near a joint, patients might also experience swelling of the joint or a reduced range of motion. Typically, constitutional symptoms are not present. According to Turcotte et al.’s study [17] involving 70 patients, the average duration of symptoms for individuals with chondroblastoma was reported to be 20 months.

Treatment usually involves extended intralesional curettage [7], and local adjuvant therapies such as cryotherapy, phenol, argon, hydrogen peroxide, cryotherapy, and adjuvant radiotherapy are employed to reduce the risk of recurrence [7,18,19]. In primary aggressive cases, an alternative option is to perform either a marginal or wide resection [5]. Despite these interventions, local recurrence rates of up to 35% have been reported in the literature [20]. Although rare, chondroblastoma metastasis has been documented, most commonly affecting the lungs, although metastasis to soft tissue and bone has also been reported. Metastatic chondroblastoma presents a significant management challenge, often requiring aggressive surgical intervention and systemic therapies [21]. Despite advances in diagnostic and therapeutic modalities, the molecular mechanisms underlying chondroblastoma metastasis remain poorly understood. Additionally, there is a lack of evidence regarding the best treatment options for metastasis due to chondroblastoma, with previous studies demonstrating a wide heterogeneity of treatment approaches, highlighting the importance of further research in elucidating the factors driving tumor dissemination [22]. A deeper understanding of the pathogenesis and optimal management strategies for chondroblastoma is essential to improving patient outcomes and minimizing the risk of recurrence and metastasis.

## 2. Chondroblastoma Metastasis

### 2.1. Chondroblastoma Metastasis: A Rare Occurrence

While typically considered benign, chondroblastoma is classified in the “intermediate, rarely metastasizing” category according to the 2013 World Health Organization classification of bone tumors [23]. The phenomenon of metastasis can be categorized into three distinct clinical scenarios based on its temporal occurrence and relationship to the primary tumor. These scenarios are as follows:

- The most prevalent scenario entails concurrent metastasis with local tumor presence, illustrating a synchronous development pattern [24,25,26,27,28,29,30,31,32].
- Metastasis manifesting concomitantly with the initial presentation of the primary tumor, indicating an early dissemination propensity [33,34,35,36,37,38].
- The occurrence of late metastasis in the absence of local recurrence, highlighting a delayed dissemination process [2,39,40,41,42,43,44,45,46,47].

These categorizations facilitate a deeper understanding of the metastatic process, enabling more tailored and effective therapeutic strategies.

Metastasis, although rare (1%), can occur in chondroblastoma, affecting the lung, bone, and soft tissue [12]. However, such cases typically exhibit indolent growth and do not significantly impact mortality. Nonetheless, isolated cases of fatal outcomes due to massive local recurrence have been documented, and spontaneous malignant transformation without radiation therapy has also been reported [22,48]. Metastasis can occur at the time of diagnosis or emerge after a period ranging from 1 to 2 years to 33 years following the initial diagnosis [24,25,26,30,42,44,45,46,47]. Unfortunately, histologic criteria for predicting the risk of local recurrence or metastases remain unreliable, including cytologic atypia, mitotic rate, the presence of giant cells, or lymphovascular invasion [42]. A study conducted by Lin, P.P. et. al. [49] determined the factors that contribute to local recurrence and metastasis in chondroblastoma. The findings of their investigation suggested that the recurrence of chondroblastoma primarily hinges on two key factors: incomplete surgical intervention and the tumor’s biological aggressiveness. Among the patients treated, the biologically aggressive nature of the tumor, as evidenced by malignant transformation, was responsible for three out of four instances of recurrence. This malignancy was evident in both histological characteristics and clinical progression. Despite receiving intensive treatment involving chemotherapy and extensive surgical procedures, all three patients developed metastases and succumbed to the disease. The designation of malignant chondroblastoma remains contentious, as consensus regarding its diagnostic criteria is lacking. While the exact mechanisms of chondroblastoma metastasis are unclear, factors like tumor size, histological characteristics, and genetic changes may play a role [9,50]. Metastasis can significantly influence treatment choices and patient prognosis, requiring close monitoring. Several case studies were conducted to understand chondroblastoma metastasis more rigorously and with a large cohort. The pain was an almost constant symptom among all the patients, and about 80% of the tumors were in long bones, like the humerus, tibia, and femur [51]. A minority affected the flat bones and short tubular bones of the hand and foot, with a peculiar affinity for the calcaneus and talus [52]. The prognosis depends basically on a relatively high rate of recurrence, sometimes with local seeding of soft tissues and joint space. Such recurrences require wide resection with arthrodesis or even amputation. A study found that recurrence was observed when curettage was incomplete or when tumor cells were disseminated during surgery [53]. Prashant Narhari et al. [54] documented a case of chondroblastoma recurrence leading to osteosarcoma, emphasizing the need for vigilant monitoring and aggressive intervention.

### 2.2. Molecular and Genetic Causes of Metastasis

In recent years, significant advancements have been achieved in understanding the genetic characteristics of chondroblastoma, giant cell tumors of bone [55,56,57,58,59]. New research has found that various tumors frequently experience mutations in their epigenetic regulatory genes, leading to abnormal gene expression patterns [57]. This can result in the suppression of tumor suppressor genes and the activation of oncogenes, ultimately promoting the development of cancer [60]. These tumors share common driver mutations, particularly in genes encoding histone H3.3 (H3F3A and H3F3B), leading to widespread epigenetic alterations [58]. These mutations have facilitated the development of advanced diagnostic tools that distinguish between different tumor types containing giant cells. A recent study has even discovered mutations in histone H3.3 in chondroblastoma [58,59,61]. These mutations are primarily found in the H3F3B gene (occurring in 95% of cases, with 93% of those being H3F3B and 7% H3F3A mutations), and they exclusively exhibit the K36M point mutation in both H3F3A and H3F3B [58]. These mutation-induced epigenetic changes significantly influence cellular differentiation and function. While these discoveries offer enhanced precision in tumor classification and prognostication, further research is needed to fully understand the complex relationship between epigenetic modifications and tumorigenesis. Future investigations aim to uncover the underlying biological mechanisms driving tumor development and progression, potentially revolutionizing diagnostic and therapeutic approaches and improving patient outcomes in these challenging neoplasms. In a recent study, researchers assessed the expression of the K36M-mutated protein using immunohistochemistry in various tumors. The study found that 95% of chondroblastomas carry the point mutation K36M in either the H3F3A or H3F3B genes [62]. Out of 1894 tumors studied, including 85 chondroblastomas and 10 clear-cell chondrosarcomas, the mutated protein was expressed in 82 chondroblastomas and 1 clear-cell chondrosarcoma known to harbor the mutation [63]. Moreover, three chondroblastomas and nine clear-cell chondrosarcomas without the H3F3 mutation showed no p.K36M mutation expression. Additionally, 1799 other tumors, including 1047 primary bone tumors and 507 soft tissue and joint tumors, were negative for the mutation, indicating the specificity and sensitivity of this immunomarker as a diagnostic tool for chondroblastoma [63]. In contrast, tumors without the mutation did not exhibit any immunoexpression of K36M. Similarly, Cleven et al. [64] reported mutations in histones H3F3A and H3F3B that seem to be highly specific but less sensitive tools for distinguishing between giant cell tumors of bone and chondroblastoma compared to other giant cell-containing tumors, suggesting a potential role of methylation at this residue in the development of the disease.

Moreover, the high frequency of these mutations implies their significant role in tumorigenesis and their potential as targets for therapeutic strategies. Further research is needed to better understand the risk factors and mechanisms underlying chondroblastoma metastasis and to improve outcomes for affected individuals.

## 3. Metastatic Chondroblastoma: Clinical Signs and Diagnostic Criteria

### 3.1. Patterns and Sites of Metastasis in Chondroblastoma

Understanding the patterns and sites of metastasis in chondroblastoma is crucial for accurate diagnosis, treatment planning, and prognostication. While chondroblastoma is primarily considered a benign bone tumor, it can infrequently exhibit metastatic behavior, spreading beyond its primary site to distant locations such as bone, lung, soft tissues, and liver. Here, we explore the patterns and common sites of metastasis observed in chondroblastoma cases.

#### 3.1.1. Bone Metastasis

Bone metastases may present with localized pain, swelling, or pathological fractures. Imaging modalities such as bone scans, magnetic resonance imaging (MRI), or positron emission tomography–computed tomography (PET-CT) scans are valuable for detecting and assessing the extent of bone metastases. In a study by Murphy et al. [65], it was found that bone metastases can develop from conventional chondroblastoma of the rib, even 11 years after the primary tumor has been removed. The metastases remained confined to the bone and did not involve the lungs, representing a distinct pattern of metastatic spread in chondroblastoma [65]. A study on 495 chondroblastoma patients found that the femur, talus, and calcaneum were the most commonly affected bones [66]. Most patients were under 30 years old, and tumors, primarily located in the medulla, can exhibit recurrence and metastasis even when appearing benign [66]. Birch et al. [30] documented a case study where a primary chondroblastoma originating from the third rib metastasized to the skull, acetabulum, and scapula 23 years after initial diagnosis. A rare case of chondroblastoma, typically benign but with metastasis to the mandible, underscores the need for vigilance in diagnosis and treatment, prompting scapula resection due to disease progression [42]. Systemic spread is a potential complication in these patients, which is difficult to treat and can be a vital threat. In a study by Baumhoer et al. [26], a chondroblastoma initially located in the acromion metastasized to T5, the third rib, and the craniovertebral junction, challenging the traditional classification of chondroblastomas as rarely metastasizing. According to a study by Ramappa et al. [67], metastasis occurred in the skeletal region, particularly in the proximal part of the tibia and the proximal part of the humerus.

#### 3.1.2. Pulmonary Metastasis

The lungs represent one of the most common sites of metastasis in chondroblastoma [36]. Pulmonary metastases often manifest as multiple nodules or masses detected on chest radiographs or computed tomography (CT) scans. Patients with pulmonary metastatic chondroblastoma may present with respiratory symptoms such as cough, dyspnea, or chest pain. The mechanism of pulmonary metastases in chondroblastoma was explored by Ozkoc et al. [46]. While it is postulated to involve vascular invasion, instances of pulmonary metastasis before surgery challenge this theory, indicating alternative pathways may be at play. Radiographically, pulmonary metastases exhibited diverse presentations, including nodules without calcification and cystic, cavitary nodules [37]. Notably, pulmonary metastases lacked histological features suggestive of malignancy but still posed significant risks, leading to complications such as secondary pneumothorax and death in documented cases. In a report by Wing et al. [36], a particularly uncommon instance of locally recurring chondroblastoma with pulmonary metastases was detailed. After the initial resection of the tibial chondroblastoma, the patient experienced palpitations, a dry cough, and difficulty breathing four years later. A chest CT scan revealed multiple pulmonary soft tissue nodules with calcification, and robotic-assisted bilateral pulmonary wedge resections were carried out, followed by denosumab therapy. Subsequent imaging confirmed that the treatment was successful, with no signs of tumor recurrence. In a case report by Tamura et al. [37], a patient was diagnosed with bilateral pneumothorax due to pulmonary metastasis of a chondroblastoma, a rare occurrence. The patient had undergone surgery for a chondroblastoma in the right ischium 20 months prior. The author suggested chest CT imaging for detecting hidden metastases, highlighting the importance of investigating the possibility of lung metastasis in patients with a history of malignancy and bilateral pneumothorax. These cases highlight the importance of ongoing surveillance for pulmonary metastasis in chondroblastoma patients to enable timely intervention and emphasize the need to consider metastatic disease in patients with a history of chondroblastoma when pulmonary nodules are detected.

#### 3.1.3. Soft Tissue Metastasis

In rare instances, chondroblastoma may metastasize to soft tissues adjacent to the primary tumor site or distant soft tissue locations. Metastasis of chondroblastoma to soft tissue is a significant clinical occurrence that can appear in various parts of the body. The soft tissue involvement can be classified into two categories: muscles and cutaneous sites, such as the nose and scalp. Accurately identifying these distinct patterns of metastasis is vital for proper diagnosis, treatment planning, and predicting outcomes for patients with chondroblastoma. Soft tissue metastases may present as palpable masses or nodules, often requiring histological evaluation for a definitive diagnosis [68]. In a case reported by Khalli et al. [31], metachronous soft tissue metastases from a primary rib chondroblastoma were documented, involving various body parts but notably sparing the lungs over a 17-year period post-resection. The findings across multiple studies highlighted the uncommon but notable potential for chondroblastoma to undergo metastasis, challenging previous perceptions of its behavior. Elek et al. [29] documented a case where a chondroblastoma originating from the calcaneus metastasized to the scalp and nose, with chemotherapy showing significant efficacy against lung lesions. Baumhoer et al. [26] described chondroblastoma in a 54-year-old patient with the acromion as the primary site, which metastasized to soft tissue, lung, and bone. Schajowicz et al. [69] observed an atypical progression of chondroblastoma from the distal head of the second metatarsus to the thigh and buttock, while Seline et al. [40] reported on a case with cutaneous metastases from a primary lesion in the vertebral column, initially presenting with unilateral hyperhidrosis. Lastly, Reyes et al. [70] noted a case of scalp and nose metastasis, showcasing aggressive and recurrent tumor behavior over 30 years, eventually classifying it as a malignant neoplasm. 

#### 3.1.4. Liver and Regional Lymph Node Involvement in Chondroblastoma Metastasis

Sirsat et al. [48] documented the initial case of spontaneous malignant transformation in a chondroblastoma patient. After eight years of radiologic evidence of the tumor and six years post-initial curettage, the development of an anaplastic tumor led to the patient’s demise, with suspected liver metastases. In a report by Kahn et al. [22], a case involving a patient diagnosed with chondroblastoma and subsequent metastasis was documented. The patient’s follow-up revealed metastases to the lungs, liver, soft tissue, and diaphragm, ultimately resulting in the patient’s demise.

Regional lymph node involvement is believed to be infrequent in individuals with chondrosarcoma, yet its precise prevalence remains uncertain. Although uncommon, metastatic dissemination to regional lymph nodes has been documented as a prognostic indicator in cases of chondroblastoma [71]. Regional nodes may serve as a prognostic indicator in chondroblastoma.

Overall, the patterns and sites of metastasis in chondroblastoma can vary, with pulmonary metastases being the most common. However, metastatic chondroblastoma cases may exhibit heterogeneous clinical presentations, necessitating a comprehensive evaluation and a multidisciplinary approach to diagnosis and management.

### 3.2. Diagnosing Metastatic Chondroblastoma

Diagnosing metastatic chondroblastoma presents several challenges due to its rarity and the similarity of its clinical and radiological features to other bone tumors and metastatic lesions. The differential diagnosis of metastatic chondroblastoma encompasses a broad spectrum of bone neoplasms and metastatic diseases, necessitating a comprehensive approach to achieve accurate diagnosis and appropriate management.

#### 3.2.1. Histopathological Examination

The definitive diagnosis of metastatic chondroblastoma relies on the histopathological examination of biopsy specimens obtained from metastatic lesions. It is composed of mononuclear cells with nuclear grooves and osteoclast-type multinucleated giant cells scattered throughout [72]. Although chicken-wire calcification is often present, it may not always be visible in decalcified samples. Osteoclast-type multinucleated giant cells are consistently present, and no predictive histological features for recurrence have been identified [73]. Clear-cell chondrosarcoma is characterized by large neoplastic cells with clear and slightly eosinophilic cytoplasm, sometimes resembling conventional chondrosarcoma [72]. Differentiating chondroblastoma from giant cell tumors can be difficult, particularly in cases where chondroblastomas contain numerous giant cells and a minimal chondroid matrix [74]. However, factors like epiphyseal location, lytic radiographic appearance, and occurrence in younger patients with open epiphyseal plates can aid in distinguishing chondroblastomas from giant cell tumors. However, distinguishing metastatic chondroblastoma from other bone tumors with similar histological features, such as chondrosarcoma or giant cell tumor of bone, can be challenging and may require ancillary studies [3].

#### 3.2.2. Immunohistochemical Analysis

Immunohistochemical staining can aid in differentiating metastatic chondroblastoma from other bone tumors. In a comprehensive review of chondroblastoma studies, it was found that immunohistochemistry (IHC) staining, as observed in various investigations, notably highlighted the utility of S100P and DOG1 markers [75]. These markers exhibited positivity in a significant percentage of cases, aiding in confirming the diagnosis of chondroblastoma and distinguishing it from other bone tumors like giant cell tumors. Additionally, SOX9, another immunomarker, consistently demonstrated positive expression across chondroblastomas in various studies, further solidifying its role in diagnosis. Recent studies have discovered that 95% of chondroblastomas possess a point mutation K36M mutation in histone H3.3, as showcased through immunohistochemistry in a broad analysis of 1894 tumors. This finding underlines the potential of the K36M-mutated protein as a highly specific and sensitive diagnostic marker for chondroblastoma [58,59,62,63]. These findings collectively underscore the importance of immunohistochemical analysis in accurately characterizing chondroblastomas and guiding appropriate treatment strategies. However, it is not very specific in distinguishing chondroblastomas from other chondroid tumors, particularly chondromyxoid fibroma [76]. The interpretation of immunohistochemical results must be considered in conjunction with histological findings and clinical context [77].

#### 3.2.3. Radiographic Imaging

Radiographic imaging, including plain radiography, computed tomography (CT), magnetic resonance imaging (MRI), and bone scintigraphy, plays a crucial role in the evaluation of metastatic chondroblastoma [78]. Radiographic features of metastatic chondroblastoma may include lytic lesions with well-defined borders, cortical thinning or destruction, and soft tissue extension. In a retrospective analysis conducted by Blancas C et al. [79], 18 patients were diagnosed with chondroblastoma and revealed distinct patterns in the localization of the tumor, with a clear preference for the distal femur, proximal humerus, and ilium. The radiographic evaluation uncovered characteristic features like calcification of the tumor matrix in 50% of cases and periosteal reactions in 44%. Magnetic resonance imaging (MRI) consistently depicted a homogeneous intramedullary lesion. The tumor demonstrated isointensity to muscle on T1-weighted sequences and heterogeneous signal intensity on T2-weighted sequences. These findings emphasize the importance of recognizing chondroblastoma in young patients with osteolytic epiphyseal lesions and highlight the usefulness of imaging techniques like CT and MRI in characterizing tumor extension.

## 4. Treatment Approaches for Metastatic Chondroblastoma

Managing metastatic chondroblastoma poses unique challenges due to its rarity and the potential involvement of multiple organ systems. Treatment strategies aim to control tumor growth, alleviate symptoms, preserve function, and improve the overall quality of life [80]. While no standard guidelines exist for metastatic chondroblastoma, therapeutic decisions are typically individualized based on factors such as tumor burden, extent of metastasis, the patient’s overall health, and treatment goals. Here, we elaborate on the various treatment modalities employed in managing metastatic chondroblastoma.

### 4.1. Surgical Treatment

Surgical resection stands as one of the crucial components among the treatment modalities for metastatic chondroblastoma [7], particularly for localized lesions amenable to surgical excision. When feasible, complete resection of metastatic lesions aims to achieve local disease control, alleviate symptoms, and prevent further progression. However, the surgical approach may be limited by extensive metastatic involvement or lesions in critical anatomical locations. A comprehensive review of metastatic chondroblastoma cases highlights the diverse clinical presentations and treatment modalities. Kunje et al. [44] documented a 33-year-old male patient with metastasis to both lungs who underwent hemipelvectomy without recurrence. In Kahn et al.’s [22] study, a 13-year-old male patient experienced multiple lung metastases and recurrences, which were managed with curettages and partial resections. Similarly, Kyriakos et al. [34] reported a 9-year-old male with lung metastases and one recurrence, treated with curettage and chemotherapy. Wellman et al. [47] reported a case of chondroblastoma treated with surgical resection, involving a 29-year-old male patient. The patient presented with a large chondroblastoma in the scapula, necessitating forequarter amputation after 12 years of tumor growth. Ultrastructural examination revealed characteristic features of chondroblastoma cells, including indented, multilobular nuclei, subdivision of cytoplasm, intracytoplasmic fibrils, and multinucleated giant cells. Additionally, the interstitium exhibited collagen and reticular fibrils, potentially originating from the tumor cells. Riddell R. J. et al. [32] described a case study of a 14-year-old female patient who had been diagnosed with metastatic chondroblastomas. The metastases were found in the left lung and affected both lobes. Two distinct lesions were observed in a subpleural location. The treatment modalities used were curettage and subsequent amputation. In another study, Green and Whittaker et al. [24] documented the clinical presentation of a 13-year-old female patient with chondroblastoma metastasis. The metastases were identified in both lungs, with several occurrences noted. The chosen treatment approach for managing these metastatic lesions involved curettage. Huvos et al. [25] reported on a 16-year-old male patient with chondroblastoma metastasis to both lungs, characterized by multiple recurrences, and managed with curettage. Van Horn et al. [27] reported late pulmonary metastases in a patient treated with curettage and bone grafting for distal femur chondroblastoma. After a pathologic fracture and local recurrence, the patient underwent radical resection and knee arthrodesis. Despite pulmonary metastases, metastasectomy resulted in no tumor recurrence at the latest follow-up, indicating successful management.

Khalili et al. [31] presented a case of a 43-year-old male with chondroblastoma metastases to various soft tissue sites, including the scalp, neck, paraspinal region, forearm, and buttock. Despite multiple recurrences, treatment primarily involved resection of the metastatic lesions. Seline et al. [40] reported a case of a 35-year-old male with chondroblastoma metastasis to the scalp, undergoing multiple recurrences treated with resection and radiotherapy.

Concerning the spread of metastases, there are a few documented fatal cases of chondroblastoma showing pulmonary metastasis of the same histologic characteristics as the removed tumor [25,34], thus presenting a metastatic chondroblastoma. In a recent study by Yonghui Wu et al. [81], surgical resection via video-assisted thoracoscopy surgery (VATS) proved effective in managing chondroblastoma in the rib, highlighting its comparable efficacy to traditional open thoracic surgery and minimizing patient injury. 

### 4.2. Palliative Care

Palliative care plays a vital role in the comprehensive management of metastatic chondroblastoma, focusing on symptom management, psychosocial support, and improving the quality of life for patients and their families. Palliative interventions may include pain management, symptom control, psychological support, and end-of-life care planning [82].

#### 4.2.1. Radiation Therapy

Radiotherapy serves as a viable treatment modality for individuals deemed unsuitable candidates for surgery, particularly those with recurrent or unresectable chondroblastoma [83]. Its utilization aims to mitigate the risk of recurrence; however, it is not considered a conventional treatment approach. Notably, radiotherapy is not advocated following a complete tumor resection due to the potential development of radiation-induced chondrosarcoma. The available literature offers limited insight into the radiation sensitivity of chondroblastomas.

Radiation therapy may be utilized as an adjunctive treatment modality for metastatic chondroblastoma, especially for unresectable lesions, or as palliative therapy to relieve pain and control local disease progression [84]. However, one of the studies suggested that radiation and chemotherapy have no current role in the treatment of chondroblastoma [85]. According to a case study conducted by David Dahlin and colleagues [39], radiation therapy is not recommended for typical chondroblastoma due to the risks associated with irradiation and the usually favorable outcomes achieved with conservative surgery. Radiation therapy can be considered and might provide a cure for patients when a surgical option is not feasible. Zhang et al. [86] presented a successful surgical approach for managing chondroblastoma in the talus, demonstrating no recurrence or metastasis during the follow-up. A study conducted by Wirman et al. [45] revealed that a moderate palliative dose of radiation (4000 rads over four weeks) reduced the size of the tumor by only 50%. While this response was not evident six weeks after treatment, it became apparent six months later. The study also observed occasional lesions that displayed aggressive tendencies, resulting in clinical recurrences and even metastasis over the course of 34 years. Nonetheless, Dahlin et al. [39] reported two cases from the Mayo Clinic series in which radiation yielded satisfactory outcomes.

#### 4.2.2. Systematic Treatment

Various chemotherapy drugs have been utilized in several studies for the systemic therapy of chondroblastoma metastasis. These treatments aim to address the challenges posed by the metastatic spread of chondroblastoma, offering potential avenues to manage and control the disease beyond localized approaches. Additionally, emerging therapies like denosumab have shown promise in treating chondroblastoma metastases, providing new insights into the management of this rare condition. Baumhoer et al. [26] reported a case of chondroblastoma with pulmonary metastasis, treated initially with palliative doxorubicin chemotherapy, showing stable disease after four cycles but halted due to treatment-related fatigue. Subsequent evaluation revealed progressing pulmonary lesions and an MSH2 mutation. Experimental immunotherapy with pembrolizumab was initiated, resulting in a slight decrease in selected pulmonary nodules and bone lesions, indicating an overall stable disease despite the inconsistent immunophenotyping of mismatch repair proteins. Binesh et al. [43] reported a fatal case involving a 9-year-old boy who had metaphyseal chondroblastoma of the tibia. The primary lesion was surgically treated, but unfortunately, it led to the development of pulmonary metastases. After surgery, the patient received a single round of chemotherapy that included doxorubicin, vincristine, and cyclophosphamide. However, despite the treatment, the patient did not respond well, and the chemotherapy was stopped. A case study by Kyriakos et al. [34] documented a patient with chondroblastoma whose condition unfortunately led to pulmonary metastases before any surgical intervention for the primary tumor. The treatment plan included systemic chemotherapy, which began with a 12-week course of vincristine, followed by two additional biweekly cycles of vincristine and cyclophosphamide. Despite these efforts, the lung nodules and recurrent tibial tumor continued to grow. As a result, chemotherapy was discontinued after the patient received a cumulative dosage of vincristine and cyclophosphamide. Ostrowski et al. [28] documented an uncommon instance of malignant chondroblastoma in a 47-year-old male, emerging as a recurrent tumor 18 years post-wide excision of a conventional pelvic chondroblastoma. Despite undergoing chemotherapy and radiation, the patient succumbed to the disease within eight months of the initial presentation.

### 4.3. Denosumab: A Novel Therapeutic Approach

While surgical resection is the mainstay of treatment, recurrence rates remain a concern, particularly in cases where complete excision is challenging. In recent years, there has been growing interest in exploring alternative treatment modalities to improve outcomes for patients with chondroblastoma [87,88]. One such approach involves using denosumab, a monoclonal antibody that targets the RANK ligand pathway, which plays a crucial role in osteoclast-mediated bone resorption [89,90]. Denosumab is a fully human monoclonal antibody that binds to the RANK ligand (RANKL), thereby inhibiting its interaction with RANK receptors on osteoclast precursors and mature osteoclasts [91]. RANKL plays a crucial role in the interaction between human bones and the immune system. It binds to RANK receptors on osteoclasts and their precursors, essential for their development and function. This binding activates several pathways, ultimately turning on the expression of NFATc1, vital for transforming osteoclast precursors into functional osteoclasts and managing genes involved in their formation and survival [92,93,94]. It is now clear that the RANKL/RANK axis affects not only osteoclasts but also tumor cells. Research has shown that the RANKL/RANK pathway in cancer cells is linked to tumor growth and the advanced stages of the disease [95]. Denosumab is widely used not only to treat osteoporosis but also to prevent bone complications from metastases in solid tumors like breast and prostate cancer [92,93]. The therapeutic approach has demonstrated efficacy in managing various bone lesions characterized by the presence of osteoclast-like giant cell tumors. This includes, but is not limited to, central giant cell tumors, giant cell granulomas, aneurysmal bone cysts, fibrous dysplasias, osteoblastomas, and chondroblastomas [96]. Chondroblastomas typically contain multinucleated osteoclast-like GCs, and some evidence suggests that these GCs may play a key role in the pathogenesis of chondroblastoma via the RANK/RANKL pathway. Huang et al. [97] demonstrated that RANKL is expressed in chondroblastoma tumor cells and may play a role in osteolytic bone destruction. Another study demonstrated the expression of RANK, RANKL, and osteoprotegerin in chondroblastomas [96]. Using RNA in situ hybridization, Suster et al. [98] observed that RANKL RNA is expressed in 92% of the 26 chondroblastoma lesions. Thus, the evidence from the literature mentioned above for the expression of RANKL in chondroblastoma makes this tumor a suitable target for denosumab treatment. Marco Focaccia et al. [37] presented a case study investigating denosumab treatment’s effectiveness in controlling chondroblastoma metastases. The study demonstrated the safety of this approach in an adolescent patient over nearly two years. This case underscores the efficacy and safety of denosumab in managing metastatic chondroblastoma, offering a promising therapeutic avenue for patients with progressive disease. Similarly, a study by Nicholas Calvert et al. [99] described cases of chondroblastoma treated with denosumab. The patient reported no known adverse effects throughout denosumab therapy, suggesting its tolerability and potential efficacy in preventing tumor recurrence. Similar findings were also observed by Visgauss et al. [100], who reported the positive clinical response of denosumab along with its impact on cellular RANK/RANK ligand activity, suggesting that denosumab could be considered in the treatment of chondroblastoma. These cases highlight the complexities of managing chondroblastoma and emphasize denosumab’s role as potential adjuvant therapy and for the treatment of metastatic chondroblastoma.

**Figure 1:**
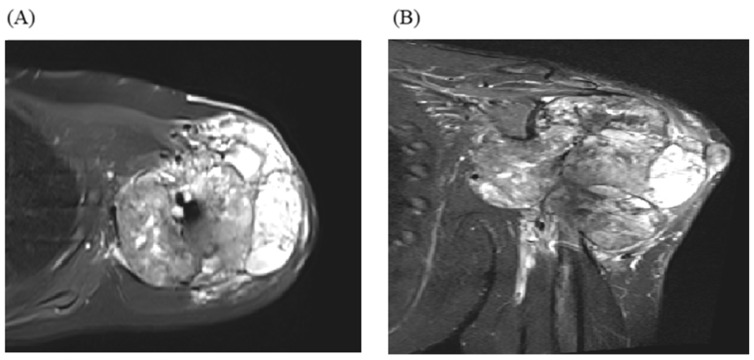
(**A**) Axial magnetic resonance imaging (MRI) with T2-weighted fat suppression and a coronal image (**B**) revealing heterogenous signal intensity with an articular extension.

**Figure 2:**
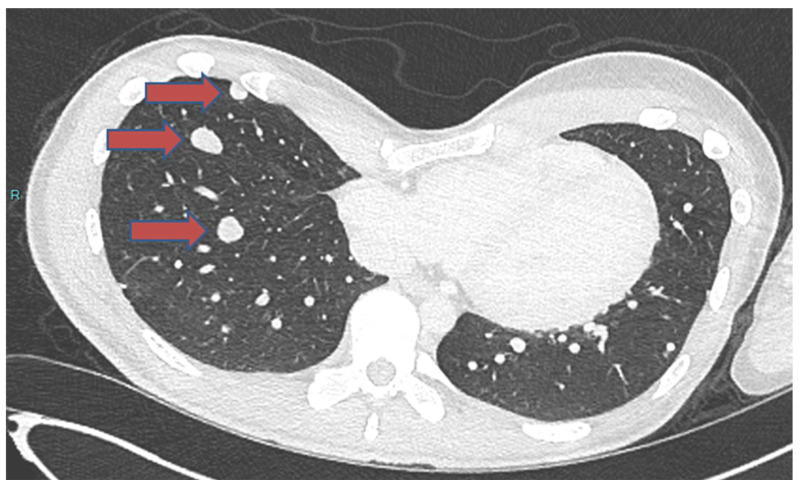
A chest CT scan showing the progression of metastasis before denosumab treatment. Red arrows indicate the nodules.

**Figure 3:**
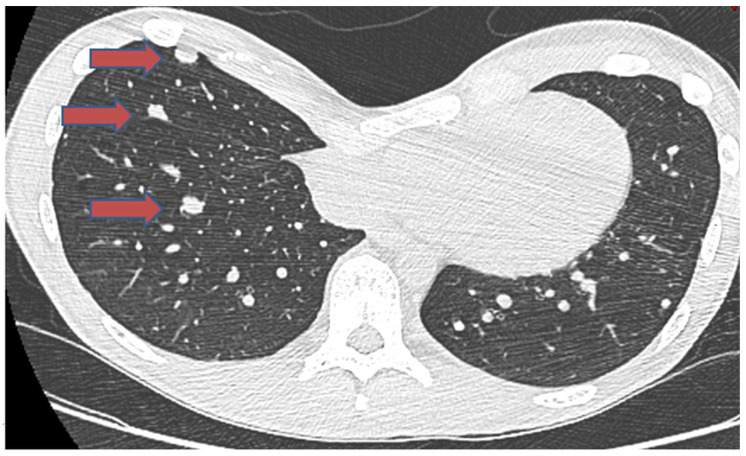
A chest CT scan, one year after denosumab treatment, showing a reduction in the size of 3 nodules and calcification marked by red arrows.

In a recently published case study [93], a 19-year-old male patient was diagnosed with chondroblastoma of the proximal humerus, concurrently exhibiting localized lung metastases (Figure 1). The treatment plan included a wide local excision of the tumor and shoulder arthrodesis. The surgical approach involved utilizing a vascularized fibular graft and a tibial allograft. Additionally, the surgical team removed the lung metastases. However, despite the initial intervention, the disease continued to progress. The patient developed bilateral lung metastases. Subsequently, a course of denosumab was administered to the patient (Figure 2). This treatment proved highly effective in controlling the metastatic progression and preventing local disease recurrence. The clinical efficacy of denosumab was observed 24 months postoperatively and 13 months after the commencement of denosumab treatment (Figure 3).

## 5. Survival Outcome in Chondroblastoma Metastasis

Several case studies have shed light on the survival rates of patients with chondroblastoma metastasis. Ozkok et al. [46] reported a study of a 53-year-old male with lung metastasis who succumbed to the disease after the study was published. Conversely, Kahn et al. [22] documented a 13-year-old male who survived for six years after developing metastases in the lung, liver, diaphragm, and subcutaneous tissue. Huvos et al. [25] reported the case of a 16-year-old female with lung metastasis who remained alive after the metastatic event. In contrast, Wirman et al. [45] observed a 38-year-old male with lung metastasis who survived for nine months post-metastasis. Binesh et al. [43] noted early mortality after lung metastasis in a 9-year-old male. The survival duration after lung metastasis varied among other cases, including those reported by Kyriakos et al. [34] (4 years and 9 months), Ostrozski et al. [28] (9 months), and Reyes et al. [70] (6 months). Additional cases with lung metastasis, such as those documented by Suneja et al. [2] and Sirsat et al. [48], have varied survival outcomes, with some not reported. Notably, Schajowicz et al. [69] reported a 32-year-old female with subcutaneous metastasis who survived for six years post-metastasis. Overall, these case studies underscore the heterogeneity in survival outcomes among patients with chondroblastoma metastasis, highlighting the need for further research into prognostic factors and treatment strategies.

Table 1 compiles comprehensive information on patients with histologically confirmed chondroblastoma metastases, as reported in the literature. The table includes crucial details such as patient age, sex, tumor location, symptoms, recurrence, metastasis occurrence, histology, interval to metastasis, follow-up, survival status, and treatment approaches. Notably, some cases exhibit benign metastases, while others are malignant. The cases exhibited diverse localization patterns, affecting anatomical sites including the pelvis, proximal humerus, femur, distal femur, tibia, rib, scapula, calcaneum, talus, spine (L4), and soft tissue. Treatment strategies varied widely, ranging from surgical interventions such as curettage, resection, and amputation to adjuvant therapies like radiation therapy and denosumab treatment. Multiple studies have reported cases of chondroblastoma metastasis, and some patients experienced tumor recurrence. To manage chondroblastoma metastases, surgical approaches, including curettage, resection, and amputation, were mostly employed.

## 6. Conclusions

Chondroblastoma is a rare primary bone tumor, usually found in the epiphyseal areas of long bones, with susceptibility to local recurrence and uncommon metastasis. Though considered benign, controlling local and metastatic chondroblastoma requires a comprehensive approach. The primary treatment for chondroblastoma involves surgical intervention. Metastasis, although uncommon, mainly to the lungs, further complicates management and necessitates aggressive surgical and systemic interventions. Recent advancements in understanding chondroblastoma’s genetic and molecular drivers, have provided new insights into its pathogenesis and potential therapeutic targets. The expression of RANKL in chondroblastoma cells has opened new avenues for treatment with denosumab, a monoclonal antibody that inhibits the RANK/RANKL pathway and shows effectiveness in controlling both local and metastatic tumors. Hence, there is a greater need to enhance our understanding of this rare tumor’s biology and refine therapeutic approaches that can enhance patient outcomes, reduce recurrence rates, and better manage metastatic cases.

## Figures and Tables

**Table 1:** Case reports with histologically proven metastases of chondroblastoma.

Author	Age/Sex	Primary Location	Site of Metastasis	Metastasis Occurrence	Histology of Bone	Interval to Metastasis	Follow-up after Metastasis	Survival Status	Treatment of Metastasis
Dahlin D.C. [39]	-	Pelvis	Lung	Late metastasis without local recurrence	Benign	58 months	-	Deceased	-
Gawlik Z. [101]	6/F	Prox. Humerus	Lung	Late metastasis without local recurrence	Benign	3 months		Deceased	Metastasectomy
Green P. [24]	13/F	Prox. femur	Lung	Occurred at recurrence	NR	20 months	10 years	Alive at final follow-up	Thoracotomy
Huvos A.G. [25]	16/F	Dist. femur	Lung	Occurred at recurrence	Benign	14 months	5 years	Alive at final follow-up	Metastasectomy
Jambhekar N.A. [35]	27/F	Talus	Lung	Occurred at initial diagnosis	Benign		4 years	Alive at final follow-up	Metastasectomy (3 surgical resection)
Joshi D.D [33]	17/F	Fibula	Lung	Occurred at initial diagnosis	Benign		3 years	Alive at final follow-up	Thoracotomy
Kahn L.B. [22]	13/M	Pelvis	Lung, liver, diaphragm, subcutaneous	Occurred at recurrence	Malignant	9 years	6 years	Deceased 6 years later	No treatment
Khalili K. [31]	43/M	Rib	Scalp, soft tissue of neck area, forearm soft tissue, buttock	Late metastasis without local recurrence	Benign	5 years	8 years	Alive at final follow-up	Resection
Kunze E. [44]	33/M	Pelvis	Lung	Late metastasis without local recurrence	Benign	12 years	6 years	Alive at final follow-up	Exploratory thoracotomy but not all nodules were removed
Kyriakos M. [34]	9/M	Prox. tibia	Lung	Occurred at the initial diagnosis	Benign		4 years and 9 months	Deceased 5 years after diagnosis	Exploratory thoracotomy, vincristine, cyclophosphamide, and doxorubicin for progression
Riddell R.J. [32]	14/F	Prox. tibia	Lung	Occurred at recurrence	Benign	34 months	9 years	Alive at final follow-up	Excision of 1 nodule
Schajowicz F. [69]	32/F	Dist. metatarsus	Subcutaneous metastasis of the gluteal region, thigh, neck	Occurred at recurrence	Malignant	5 months		Deceased 6 years after 1st diagnosis	No treatment
Sirsat M.V. [48]	15/M	Rt. Prox. tibia	Liver	Occurred at recurrence	Benign	2 years		Deceased 9 years after 1st diagnosis and shorty after metastasis	-
Sweetnam R. [102]	19/M	Prox. fibula	Lung	Late metastasis without local recurrence	Malignant	36 months	5.8 years	Alive at final follow-up	Lobectomy
Van Horn J.R. [27]	38/M	Dist. femur	Lung	Late metastasis without local recurrence	Benign	2 years	1.6 years	Alive at final follow-up	Metastasectomy
Wellmann K. [47]	29/M	Scapula	Lung	Late metastasis without local recurrence	Benign	14 years	1 year	Alive at final follow-up	Lobectomy
Wirmann J.A. [45]	38/M	Rt. acromion	Lung	Late metastasis without local recurrence	Benign	34 years.	1 year	Deceased 9 months after the diagnosis of metastasis	Refusal of treatment
Ozkoc G. [46]	53/M	Scapula	Lung	Late metastasis without local recurrence	Benign	12 years	3 years	Deceased	Palliative treatment
Baumhoer D. [26]	54/M	Acromion	Lung, T5, soft tissue (muscles), 3rd rib, craniovertevral junction	Late metastasis without local recurrence	Benign	9 years	3 years	Alive at final follow-up	Debulking surgery and radiation for bone, doxorubicin, pembrolizumab
Binesh F. [43]	9/M	Distal tibia	Lung	Late metastasis without local recurrence	Benign	2 months	Few weeks	Deceased 2 months later after surgery	Chemotherapy with doxorubicin, vincristine and cyclophosphamide
Sohn SH. [36]	21/M	L4 spine	Lung	Occurred at initial diagnosis	Benign		3 years	Alive at final follow-up	Pulmonary wedge resection
Tamura M. [41]	21/M	Ischium	Lung	Late metastasis without local recurrence	Benign	20 months	-	Alive at final follow-up	Partial metastasectomy (debulking)
Ostrowski M.L. [28]	28/M	Pelvis	Lung	Late metastasis without local recurrence	Malignant	18 years	9 months	Deceased 8 months later	Chemotherapy
Reyes [40]	32/M	Pelvis	Lung, scalp, nose	Late metastasis without local recurrence	Malignant	32 years	6 months	Alive at final follow-up	Excision of metastasis of nose, scalp
Emil M. Elek. [29]	12/M	Calcaneum	Lung, tibia soft tissue of calf	Late metastasis without local recurrence	Benign	3 months	3 years	Alive at final follow-up	Chemotherapy, metastasectomy, radiotherapy of lung
Focaccia. M. [37]	16/M	Proximal humerus	Lung	Occurred at initial diagnosis	Benign		2 years	Alive at final follow-up	Denosumab treatment
Duttaluri, R. [38]	61/F	Extraskeletal soft tissue posterolateral aspect of the left knee	Lung	Occurred at initial diagnosis	Malignant		-	Alive at time of publication	Palliative radiotherapy
Birch PJ. [30]	37/F	rib	Skull bone, acetabulum, scapula	Occurred at recurrence	Benign	23 years	3 years	Alive at final follow-up	Radiotherapy
Samargandi. [93]	19/M	Proximal humerus	Lung, shoulder metastasis to lung	Late metastasis without local recurrence	Benign	1 year 3 months	2 years	Alive at last follow-up	Partial metastasectomy and denosumab

## Data Availability

The data presented in this study are available upon request from the corresponding author.
